# Supplementary material for: Osteoporosis in Men: A Review of an Underestimated Bone Condition
Source: Int J Mol Sci. 2021 Feb 20;22(4):2105. doi: 10.3390/ijms22042105 (PMC7924179; doi:10.3390/ijms22042105)
Supplement: Supplementary file 1 [file ijms-22-02105-s001.pdf]

Authors	Type of study	Total number of subjects	Considered outcomes	Measurement	Results
**Kukuljan et al, 2011** [95]	RCT	180 men aged 50-79	To assess whether calcium-vitamin D3 fortified milk could enhance the effects of exercise on bone strength, structure, and mineral density in middle-aged and older men	Changes in BMD, bone structure, and strength at the lumbar spine, proximal femur, mid-femur, and mid-tibia measured by DXA and/or qCT	Exercise led to a 2.1% net gain in femoral neck section modulus, which was associated with an approximately 1.9% gain in areal BMD and cross-sectional area. Exercise also improved LS trabecular BMD [net gain 2.2%), but had no effect on mid-femur or mid-tibia BMD, structure, or strength. There were no main effects of the fortified milk at any skeletal site.
**Allison et al, 2013** [96]	RCT	50 older men	Investigating the influence of a 12month high impact unilateral exercise intervention on femoral neck BMD in older men.	BMD of both femurs using DXA before and after 12months of exercisen. Repeated measures ANOVA with post hoc tests.	Femoral neck BMD, BMC and cross-sectional area all increased in the exercised leg (EL) compared to the contralateral leg (CL); significant main effects of time for section modulus minimum neck width. Section modulus increased significantly in the EL but not in the CL.
**Min et al, 2020** [107]	Descriptive	6868	Association of physical activity (PA) and serum 25-hydroxyvitamin D (25[OH]D) levels with osteopenia/osteoporosis.	Physical activity (PA) divided into three groups on the base of intensity, serum vitamin D3, BMD measured by DXA	Greater osteoporosis in ascending order as follows: high PA with high 25(OH)D < moderate PA with high 25(OH)D < low PA with high 25(OH)D < high PA with low 25(OH)D < moderate PA with low 25(OH)D < low PA with low 25(OH)D**. Result consistent only in males.**
**Boonen et al, 2012** [112]	RCT	1199 men with hypogonadism-associated osteoporosis	One or more new morphometric vertebral fractures over a period of 24 months on the basis of zoledronic treatment or not.	Spine x-rays	The rate of any new morphometric vertebral fracture was 1.6% in the zoledronic acid group and 4.9% in the placebo

					group, representing a 67% risk reduction with zoledronic acid. As compared with men who received placebo, men who received zoledronic acid had fewer moderate-to-severe vertebral fractures (P=0.03) and less height loss (P=0.002).
**Orwoll et al, 2000** [113]	RCT	241 osteoporotic men.	Efficacy of 10 mg of alendronate or placebo, given daily, on bone mineral density.	Percent changes in lumbar-spine, hip, and total-body bone mineral density.	The increase in BMD in the alendronate group was greater than that in the placebo group at all measurement sites (P<0.001). The incidence of vertebral fractures was lower in the alendronate group than in the placebo group (0.8 percent vs. 7.1 percent, P=0.02). Men in the placebo group had a 2.4-mm decrease in height, as compared with a decrease of 0.6 mm in the alendronate group (P=0.02). Alendronate was generally well tolerated.
**Ringe et al, 2004** [110]	Clinical trial	134 osteoporotic men	Efficacy of treatment of Alfacalcidol (AC) combined with oral Alendronate (ALN).	BMD measured by DXA at lumbar spine and femoral neck. Spine x-rays.	AC-treated patients showed a significant mean increase of 3.5% in lumbar spine BMD, compared with a mean increase of 11.5% in men receiving ALN. The corresponding increases in femoral neck BMD were 2.3% and 5.8% for the AC and ALN groups, respectively. New vertebral fractures occurred in 24.2% of the AC-treated patients and in 10.3% of the ALN-treated patients.
**Boonen et al, 2009** [114]	Multicentric RCT	284 osteoporotic men	Efficacy and safety of 35 mg once-a-week risedronate in men with osteoporosis	Lumbar spine BMD (DXA); proximal femur BMD, bone turnover markers (BTMs), new vertebral fractures, clinical	Treatment with risedronate resulted in a significant increase from baseline to endpoint in lumbar spine BMD compared with placebo. Few new vertebral

				fractures, and adverse event (AE) assessment.	and nonvertebral fractures were reported, with no differences in fracture rates between the two groups. Significant reduction in BTMs for the risedronate group compared with placebo at all time points. No apparent differences in the pattern or distribution of AEs. Risedronate therapy was was rapidly effective as indicated by significant BTM decreases at month 3 and BMD increases at month 6 (the earliest time points tested).
**Ringe et al, 2009** [115]	RCT	316 osteoporotic men	Assessing the effect of treatment with risedronate 5 mg daily relative to control in men with primary or secondary osteoporosis over 2 years.	New vertebral fractures (x-rays) and changes in BMD at the lumbar spine, femoral neck, and total hip (DXA).	The incidence of new vertebral fractures was significantly reduced in the risedronate group at 2 years [14/152 (9.2%) for risedronate vs. 35/148 (23.6%) for control (61% risk reduction; P = 0.0026)]. Treatment with risedronate resulted in significant improvements in BMD at 2 years at all three skeletal sites. Significant reductions in the incidence of nonvertebral fractures (11.8 vs. 22.3%; P = 0.032), average loss in height, and back pain were also observed in risedronate-treated patients relative to control.
**Orwoll et al, 2012** [116]	RCT	228 osteoporotic men	Efficacy of 60 mg/ 6 months Denosumab for osteoporosis in men.	Percent change from baseline in BMD (DXA)	After 12 months, denosumab resulted in BMD increases of 5.7% at the LS, 2.4% at the total hip, 2.1% at the femoral neck, 3.1% at the trochanter, and 0.6% at the one third radius.

**Langdahl et al, 2015** [117]	Clinical trial	219 osteoporotic men	Efficacy of 60 mg/ 6 months Denosumab for osteoporosis in men.	BMD, serum collagen type I C-telopeptide, and safety.	During the open-label phase, continued BMD increases occurred with long-term denosumab treatment, resulting in cumulative 24-month gains from baseline of 8.0%, 3.4%, 3.4%, 4.6%, and 0.7%, respectively (all P < .01). The crossover group showed BMD gains after 12 months of denosumab treatment similar to those of the long-term denosumab group during the first treatment year. Significant reductions in serum collagen type I C-teleopeptide were observed after denosumab administration. Adverse event rates were similar between groups, and no new safety signals were identified.
**Nayak et al, 2017** [118]	Systematic review	4868 osteoporotic men.	Efficacy of a treatment for osteoporosis or low bone mineral density for adult men and reported fracture outcomes	PubMed, Embase, and the Cochrane Library databases were searched for relevant studies: 22 studies selected.	Lower risk of vertebral fractures with alendronate and risedronate but not with calcitonin or denosumab than in controls. For bisphosphonates as a treatment category, meta-analyses demonstrated significantly lower risk of vertebral fractures and nonvertebral fractures than in controls.
**Orwoll et al, 2003** [127]	Clinical trial	437 osteoporotic men.	Efficacy of teriparatide therapy on bone density in men with osteoporosis	BMD (DXA)	Spine BMD was greater than in placebo subjects after 3 months of teriparatide therapy, and by the end of therapy it was increased by 5.9% (20 microg) and 9.0% (40 microg) above baseline. Femoral neck BMD increased 1.5% (20 microg; p = 0.029) and 2.9% (40 microg; p < 0.001), and whole body bone

					mineral content increased 0.6% (20 microg; p = 0.021) and 0.9% (40 microg;p = 0.005) above baseline in the teriparatide subjects.
**Kaufman et al, 2005** [126]	Clinical trial	355 osteoporotic men	Efficacy of Teriparatide on vertebral fractures and bone mineral density in men with osteoporosis.	BMD (DXA)<br>Lateral thoracic lumbar radiographs	BMD gradually decreased following discontinuation of teriparatide therapy. However, the lumbar spine and total hip values remained significantly higher than baseline after 30 months of follow-up (p< or =0.001). The incidence of moderate or severe fractures was significantly reduced by 83% (p=0.01). Men who received teriparatide and who may have received follow-up antiresorptive therapy had a decreased risk of moderate and severe vertebral fractures.
**Lewiecki et al, 2018** [122]	Multicentric clinical trial	245 55-90 yr old men with osteopenia or osteoporosis	Efficacy and safety of romosozumab in treating men with osteoporosis	Percentage change from baseline in LS BMD at month 12.	At month 12, the mean percentage change from baseline in the lumbar spine (LS) and total hip (TH) BMD was significantly greater for the romosozumab group than for the placebo group. Adverse events and serious adverse events were balanced between the two groups.

Supplementary Table S1: Summary of the studies about the treatment of osteoporosis
